# Perioperative Risk: Short Review of Current Approach in Non Cardiac Surgery

**DOI:** 10.3390/jcdd12010024

**Published:** 2025-01-13

**Authors:** Andreea Boghean, Cristian Guțu, Dorel Firescu

**Affiliations:** 1 Faculty of Medicine and Pharmacy, University “Dunărea de Jos” Galați, 800008 Galati, Romania; dorelfirescu@yahoo.com; 2 Emergency Military Hospital “Dr. Aristide Serfioti” Galați, 800150 Galati, Romania

**Keywords:** risk, cardiovascular, surgery, perioperative, score

## Abstract

The rate of major surgery is constantly increasing worldwide, and approximately 85% are non-cardiac surgery. More than half of patients over 45 years presenting for non-cardiac surgical interventions have cardiovascular risk factors, and the most common: chronic coronary syndrome and history of stroke. The preoperative cardiovascular risk is determined by the comorbidities, the clinical condition before the intervention, the urgency, duration or type. Cardiovascular risk scores are necessary tools to prevent perioperative cardiovascular morbidity and mortality and the most frequently used are Lee/RCRI (Revised Cardiac Risk Index), APACHE II (Acute Physiology and Chronic Health Evaluation), POSSUM (Physiological and Operative Severity Score for the enumeration of Mortality and Morbidity), The American University of Beirut (AUB)-HAS2. To reduce the perioperative risk, there is a need for an appropriate preoperative risk assessment, as well as the choice of the type and timing of surgical intervention. Quantification of surgical risk as low, intermediate, and high is useful in identifying the group of patients who are at risk of complications such as myocardial infarction, thrombosis, arrhythmias, heart failure, stroke or even death. Currently there are not enough studies that can differentiate the risk according to gender, race, elective versus emergency procedure, the value of cardiac biomarkers.

## 1. Introduction

Worldwide, non-cardiac surgical interventions (NCS) are continuously increasing due to the aging of the population, reaching up to 85% [1,2,3]. At the same time, the mean age for the first cardiovascular event is decreasing. Patients undergoing non-cardiac surgical interventions have multiple comorbidities, and most frequently we encounter chronic coronary syndrome and stroke [2]. This category of patients has a higher risk of major perioperative adverse cardiovascular events. In a study that included adults over 45 years of age who underwent non-cardiac surgery, 1 out of 33 patients suffered a myocardial infarction, ischemic stroke or death. The highest rate of cardiovascular events was found in orthopedic, vascular and general surgeries [3,4].

**Figure 1:**
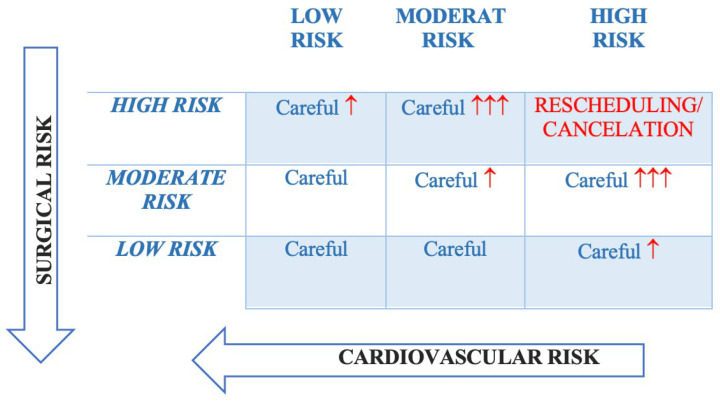
Total risk estimated by the interaction between the surgical risk and the patient’s cardiovascular risk. The red arrows signify the probability of perioperative cardiovascular complications (adapted according to the ESC 2022 guidelines).

The patient associated risk and type of surgery determines the risk of cardiovascular morbidity and mortality, so an adequate preoperative risk assessment is very important. Choosing the surgery or procedure with the lowest possible risk can decrease the total risk which should be as close as possible to the lower left corner [3,4] (Figure 1, Table 1).

Cardiovascular risk scores are necessary tools to prevent perioperative cardiovascular morbidity and mortality and the most frequently used [5] are Lee/RCRI (Revised Cardiac Risk Index), APACHE II (Acute Physiology and Chronic Health Evaluation), POSSUM (Physiological and Operative Severity Score for the enumeration of Mortality and Morbidity), The American University of Beirut (AUB)-HAS2. The Lee index created by revising the Goldman index, was designed to assess the 30-day risk of mortality, myocardial infarction, or cardiac arrest [6]. All these predictive tools can be calculated with scoring calculators online or use software systems in hospitals that automate the process based on real-time patient data.

The RCRI score comprises 6 variables:

- Creatinine ≥ 2 mg/dL = 177 mmol/L
- History of heart failure (pulmonary edema, bilateral rales, gallop, paroxysmal nocturnal dyspnea, redistribution of pulmonary circulation on chest X-ray)
- Insulin-requiring diabetes mellitus
- Intrathoracic, intraperitoneal or supra-inguinal vascular surgery
- Stroke or TIA in the past
- Ischemic heart disease (history of myocardial infarction, positive exercise test, ECG with pathological Q waves, precordial pain due to ischemia, use of nitrate medication [6] (Table 2).

The American University of Beirut (AUB)-HAS2 Cardiovascular Risk is a new risk score that has been proven superior to the RCRI (Revised Cardiac Risk Index) score [7]. It comprises 6 components:

- history of cardiovascular disease,
- angina/dyspnea,
- age ≥ 75 years,
- hemoglobin < 12 mg/dL,
- vascular intervention,
- surgical emergency.

Each criterion is given 1 point, classifying patients undergoing non-cardiac surgery into three risk groups: low (score 0–1), intermediate (score 2–3) and high (score > 3), with strong discriminative power to predict all-cause mortality, myocardial infarction, or stroke 30 days after surgery. The AUB-HAS2 score has been able to identify a large group of low-risk patients (score = 0) who generally do not require special preoperative cardiovascular evaluation or postoperative monitoring [7,8,9].

APACHE II (Acute Physiology and Chronic Health Evaluation) is a mortality estimation tool used for patients admitted to intensive care units [10]. The data used should be from the beginning of the admission (first 24 h) and should be the higher value.

The variables used to calculate the score:

- Rectal temperature
- Mean arterial pressure (mmHg)
- Heart Rate (beats/min)
- Respiratory rate (breaths/min)
- Oxygenation (PaO_2_/FiO_2_)
- Arterial pH
- Serum sodium (mmol/L)
- Serum potassium (mmol/L)
- Serum creatinine (mg/dL)
- Hematocrit (%)
- White blood cell count
- Glasgow Coma Scale (GCS)

It uses a point score based on 12 values of routine physiological measurements, age and history of severe organ insufficiency or immunocompromised status to provide a general measure of disease severity. An increasing score (range 0 to 71) indicate an increasing risk of hospital death (Table 3).

POSSUM (Physiological and Operative Severity Score for the enumeration of Mortality and Morbidity) is a scoring tool to predict morbidity and mortality based only on clinical criteria [11]. The original POSSUM was modified by researchers in Portsmouth, and it was found that it may overpredict mortality and the P-POSSUM model is now preferred.

As the name suggests, it includes two main categories:

- The physiological severity includes:

Age: <61, 61–70, >70

Cardiac signs: No failure, diuretic, digoxin, angina, hypertension medication, raised jugular venous pressure or cardiomegaly on X-ray

Respiratory: No dyspnea, dyspnea on exertion, limiting dyspnea, dyspnea at rest (rate > 30/min).

Systolic blood pressure (mmHg)

Pulse (beats/min)

Glasgow Coma Scale (GCS)

Hemoglobin (g/dL)

White cell count (×10^9^/L)

Urea (mmol/L)

Sodium (mmol/L)

Potassium (mmol/L)

Electrocardiography (Normal, Atrial fibrillation, 5 ectopic beats/min, any abnormal rhythm)

- The operative severity score include:

Operative severity (Minor, moderate, major, major+)

Number of procedures: 1, 2, 2+

Total blood loss (mL)

Peritoneal soiling: None, minor (serous fluid), local pus, free bowel content, pus or blood

Malignancy: Yes/No

Mode of surgery: Elective, emergency: Resuscitation of >2 h possible, operation within 24 h, Emergency: Immediate surgery, within 2 h.

The higher the total score, the greater the predicted risk of mortality and higher the likelihood of postoperative complications.

## 2. Summary of the Evidence

The latest guidelines of European Society of Cardiology—ESC (2022) [3], AHA/ACC American Heart Association/American College of Cardiology (2024) [4], The Thrombosis Canada clinical guides [12] and Canadian Cardiovascular Society (2017) [13] about management of patients undergoing non-cardiac surgery showed variability in definitions of low, moderate and high risk. This proves that there are gaps in the perception of risk, and currently there is no precise tool for its estimation [14].

All major guidelines recommend stepwise approach: an initial detailed history and physical examination to identify existing or undiagnosed cardiovascular diseases, followed by the assessment of surgical urgency, the type of intervention, and the estimation of overall medical and surgical risk using a validated tool, such as the Revised Cardiac Risk Index (RCRI) [14]. Pre-operative 12-lead ECG is not routinely recommended in low-risk, but it is recommended in patients who are aged ≥65 years or have known CVD, CV risk factors, or symptoms of CVD and scheduled to intermediate/high-risk NCS [15]. Patients with a risk of major adverse cardiovascular events (MACE) <1% can undergo surgery, while the rest should undergo additional investigations, depending on several factors [15].

**Figure 2:**
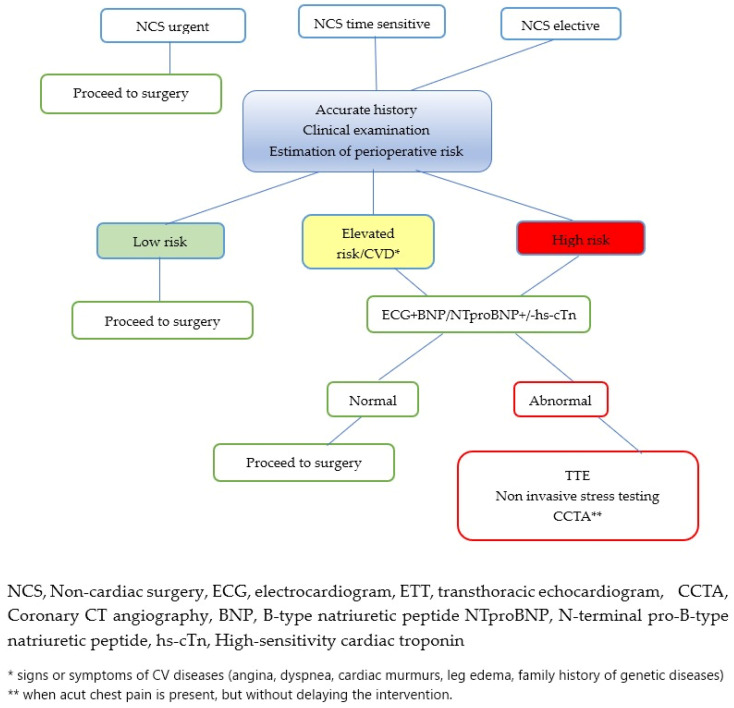
Pre-operative assessment before non-cardiac surgery.

We have developed a simplified algorithm for the rapid management of patients undergoing non-cardiac surgery, based on the timing of the surgery and the associated risk (Figure 2).

An urgent non-cardiac surgical intervention should be performed without any delay to save life or organ function and should be performed without further cardiac tests. For time-sensitive surgery, a more detailed evaluation is required, including patient history, a complete clinical examination, and the assessment of perioperative risk using a previously described prediction tool. The presence of comorbidities, age over 65 years or smoking affects the overall risk, which can be classified as:

Low risk—the patient will undergo the NCS.

Elevated risk—an electrocardiogram needs to be performed, and cardiac biomarkers need to be collected. If these investigations are normal, the patient can proceed to surgery. If the ECG and/or biomarkers are abnormal or if the patient presents signs or symptoms of CV diseases (angina, dyspnea, cardiac murmurs, leg edema, family history of genetic diseases), as in high-risk, a transthoracic echocardiography must be performed. If no significant changes are found, NCS can be performed. However, if there is left ventricular systolic dysfunction, further non-invasive stress tests are necessary. CCTA must be performed if the patient has acute chest pain but without delaying the intervention.

Regarding elective non-cardiac surgery, the same steps are followed, with all the necessary cardiac investigations performed prior to the intervention with the goal minimizing perioperative risk.

The model performance of preoperative assessment tools can be evaluated by the Area Under the Curve (AUC) of the Receiver Operating Characteristic (ROC) curve, which measures the discriminative ability of a model in predicting an outcome (such as mortality or complications). The AUC ranges from 0 to 1, with values closer to 1 indicating better performance (Table 4).

### 2.1. Biomarkers

- High-sensitivity cardiac troponin T/I (Hs-cTn T/I) quantifies myocardial injury [16],
- BNP and NT-proBNP quantifies the hemodynamic stress of the cardiac wall [17].

Hs-cTn T/I and BNP/NT-proBNP complement clinical assessment and ECG in risk prediction [18,19,20] Hs-cTn T/I and BNP/NT-proBNP concentrations are higher in patients with stress-induced myocardial ischemia [21,22].

Preoperative and postoperative levels of natriuretic peptides have been independent predictors of death or non-fatal myocardial infarction at 30 days [23]. In patients known to have cardiovascular risk factors or cardiovascular disease, or signs and symptoms of cardiac origin, it is recommended to measure BNP/NT-proBNP/hs-cTn before non-cardiac surgeries with medium and high risk [24,25].

The CCS recommends NT-proBNP or BNP testing before non-cardiac surgeries in patients who are 65 years or older, those between 45 and 64 years with significant cardiovascular disease, or those with an RCRI score ≥ 1 (Strong Recommendation; Moderate Quality Evidence) [13]. Troponin is recommended to be measured daily for 48–72 h postoperatively in patients with a >5% risk of cardiovascular events [13,26,27].

The VISION study demonstrated that an elevated level of troponin in the postoperative period was the strongest predictor of 30-day mortality. Most ischemic events remain asymptomatic and were only detected through postoperative troponin monitoring [27,28,29].

### 2.2. Echocardiography

Pre-operative FOCUS examination reduced all-cause mortality and help to triage candidates for standard TTE [30,31,32]. It is recommended for patients who present a high-risk intervention and have [33]:

- low functional capacity,
- heart murmurs,
- increased NTproBNP/BNP,
- unexplained signs and symptoms.

A preoperative diastolic dysfunction increases the risk of pulmonary edema, heart failure and myocardial infarction in the postoperative period [34].

### 2.3. Functional Capacity

Functional capacity measured in metabolic equivalents (METs), represents an essential step of the preoperative cardiac risk assessment algorithm [35] and 4 METs considered the threshold for severely depressed functional capacity [36]. It cand be measured by asking patients if they can climb 2 flights of stairs (>4 METs) or by using an instrument such as the Duke Activity Status Index (DASI), tool to assess functional capacity based on patients’ reported ability to perform a set of 12 daily activities [37].

### 2.4. Coronary CT Angiography (CCTA)

Patients who may benefit from CCTA are those that have a high-risk surgery and present with elevated perioperative cardiovascular risk, as calculated by using a validated tool (level of evidence B) [38]. It may be useful to exclude atherosclerotic plaque and obstructive CAD in patients with acute chest pain and no known CAD [39]. By contrast, no benefit of perioperative revascularization of CAD has been demonstrated; furthermore, it delayed the surgical intervention without reducing the risk of MACE [40].

### 2.5. Coronary Angiography

This investigation is not recommended routinely—it should be performed only in patients with a very high risk (level of evidence C) [41]. There are many contradictory studies regarding this topic, but none have been conducted on a large patient cohort. The indications for invasive angiography should remain the same as for patients in the non-surgical setting, and it is preferable that they be treated prior to interventions [42,43].

### 2.6. Management of Medication

#### 2.6.1. Antiplatelets

Regarding preoperative cessation of antiplatelet therapy, it is necessary to weigh in the risk of bleeding against the risk of thrombosis [44]. For patients taking aspirin as primary prevention, interruption is possible if the ischemic risk is low or moderate [45]. If the bleeding risk is low, aspirin may be continued perioperatively, but in those with a high bleeding risk, it should be stopped 7 days before the procedure and restarted within 24 h post-surgery if adequate hemostasis has been achieved [46] (Table 5). In patients who had undergone interventional revascularization and are on P2Y12 inhibitor therapy in combination with aspirin, interruption of one of these can be done after 6 months for elective PCI and at 12 months for acute coronary syndrome (ACS), thus recommending the postponement of elective surgeries [47]. If this is not possible, it is recommended that a minimum of 1 month of dual antiplatelet therapy (DAPT) is administered, with the continuation of aspirin perioperatively [47]. In patients with PCI, it has been shown that the benefit outweighs the perioperative hemorrhagic risk, recommending the continuation of aspirin at a low dose [48]. P2Y12 inhibitors in monotherapy should be interrupted 7 days prior for clopidogrel, 3–5 days prior for ticagrelor, and 7 days prior for prasugrel, only when the hemorrhagic risk is increased [49,50,51,52]. According to the American guideline, patients who are undergoing non-cardiac surgery but are within 6 months of PCI and require DAPT, can have bridging therapy with Cangrelor [53].

#### 2.6.2. Oral Anticoagulants

All guidelines, ESC, AHA/ACC, The Thrombosis Canada support that it is safe to perform surgeries with minimal bleeding risk without interrupting oral anticoagulation therapy.

In patients on vitamin K antagonists (VKA) treatment for mechanical valve prostheses, if the surgical intervention is of minor bleeding risk, it can be performed without interruption [54]. However, if the bleeding risk is high and requires an INR < 1.5, the VKA will be interrupted and overlapped with heparin [55]. If the prosthesis is in the aortic position, is a newer generation bileaflet prosthesis, and the rhythm is sinus, the VKA can be stopped without overlap [56].

In patients on VKA for atrial fibrillation or venous thromboembolism, the intervention can be performed without interrupting the anticoagulant if the risk is low [57]. If the risk is increased, warfarin will be interrupted 3–5 days prior NCS [58].

If the surgery is urgent, in patients taking VKA, the reversal of vitamin K antagonists is necessary using vitamin K, prothrombin complex concentrate, or plasma administration [59].

Direct oral anticoagulants (DOAC) available include dabigatran, apixaban, rivaroxaban, and edoxaban. Idarucizumab antagonizes the effect of dabigatran, being the only approved antagonist in urgent surgical interventions [60]. Andexanet alpha, the antagonist for the other anticoagulants, has only been studied in patients with acute hemorrhage [61].

The perioperative management of direct oral anticoagulants should be conducted based on procedural factors (the bleeding risk of the intervention, timing of the surgery) as well as patient-specific factors (bleeding history, thrombotic risk, renal function) [62,63]. We made a comparison between the approaches of the 3 guidelines, adjusted for renal function (Table 6, Table 7 and Table 8).

#### 2.6.3. Renin-Angiotensin-Aldosterone System Inhibitors (RAASi)

Angiotensin-converting-enzyme inhibitors (ACEi) and angiotensin receptor blocker (ARBs) are widely used medications for patients with hypertension and/or heart failure [64]. Preoperative interruption has been studied and found that it is more frequently associated with perioperative hypertension, while the continuation of therapy has led to hypotension [65]. Discontinuation of these drugs 24 h before surgery may be beneficial to limit intraoperative hypotension, but in patients with heart failure with reduced ejection fraction (HFrEF) perioperative continuation is reasonable [66,67]. Restarting the medication should be done as soon as possible [66,67,68].

#### 2.6.4. Beta-Blockers

If the patients are taking beta-blockers, it is recommended to maintain this treatment, as stopping it preoperatively may lead to increased mortality; this recommendation is present in all three guidelines [69]. Routine initiation of beta-blockers peri-operatively is not recommended [70], but in high risk NCS for patients who have two/more risk factors, initiation of beta-blockers at least 1 week before surgery might be beneficial [70,71].

#### 2.6.5. Calcium Channel Blockers

Treatment with calcium channel blockers (CCBs) has reduced the rates of perioperative death and myocardial infarction, but it is recommended to decrease the dosage the day of the intervention to minimize the incidence of hypotension [72]. There has been an increased mortality in patients taking dihydropyridines undergoing emergency interventions for aortic aneurysm [73].

#### 2.6.6. Statins

Statins are recommended to be continued in patients with high cardiovascular risk, while the initiation of statin therapy preoperatively is not recommended [74].

#### 2.6.7. Sodium–Glucose Co-Transporter-2 Inhibitors (SGLT-2)

This class of medications is increasingly used in patients with heart failure, diabetes mellitus, or kidney disease. It is recommended to stop the medication 3–4 days postoperatively due to the risk of euglycemic diabetic ketoacidosis [75,76].

## 3. Discussion

**Figure 3:**
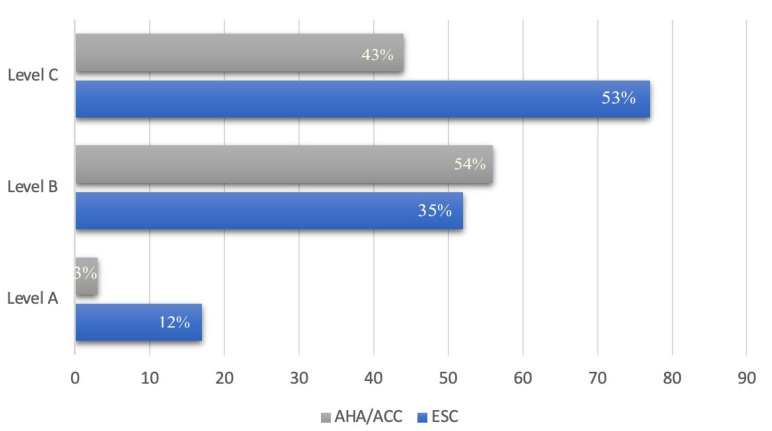
Level of evidence AHA/ACC and ESC.

The cardiovascular risk associated with non-cardiac surgeries remains an unclear area, with insufficient studies. Current guidelines, although constantly updated, still contain gaps in evidence. This is supported by the fact that the ESC guidelines contain 53% level C recommendations (consensus of the experts and/or small studies, retrospective studies, registries), while only 12% are at evidence level A (data derived from multiple randomized clinical trials or meta-analyses), and 35% at evidence level B (data derived from a single randomized clinical trial or large non-randomized studies). In the AHA/ACC guidelines, the recommendations at level A are much lower, at 3%, while the level B evidence, based on one or more randomized or non-randomized studies (75% are non-randomized), accounts for 54%. Level C evidence, based on limited data or consensus of expert opinion, totaled 43%. The CCS guidelines are based on recommendations with 48% low-quality evidence, 36% moderate-quality evidence, and 16% high-quality evidence (Figure 3). From this analysis, it appears that the guidelines’ recommendations are less evidence based and more experts’ opinion. A more precise instrument for calculating perioperative risk is necessary, one that can encompass all aspects related to both the patient and the surgical intervention. The measurement of preoperative cardiac biomarkers is a first step for the prevention of cardiovascular events, but it is still insufficiently applied. Echocardiography should be performed on all patients with high cardiovascular risk over the age of 45 for the early diagnosis of heart failure with preserved ejection fraction or CCD.

## 4. Conclusions

Surgical morbidity and mortality can be reduced or prevented through a careful preoperative evaluation, optimization of the patient’s medication, precise surgical techniques and appropriate postoperative care. Predicting postoperative outcomes using various risk assessment tools is essential, as a patient’s physiological status reflects their capacity to withstand the stress of surgery and recover without complications. Risk scores provide valuable insight into the patient’s potential for adverse events, guiding clinical decisions and helping ensure better management throughout the surgical process. We have presented the key messages of this review in Table 9.

## Figures and Tables

**Table 1:** Total CV risk.

Factors Related to the Surgical Intervention	Factors Related to the Patient
Type of surgery Urgency Immediate Urgent Time-sensitive Elective Bleeding risk	Age
	Smoking
	Thrombotic risk
	Comorbidities
	Chronic medication
	Renal function

**Table 2:** Risk of major cardiac events.

RCRI Score	Risk of Major Cardiac Events (95% CI)
0	3.9% (2.8–5.4%)
1	6.0% (4.9–7.4%)
2	10.1% (8.1–12.6%)
≥3	15% (11.1–20.0%)

**Table 3:** Hospital mortality rates.

APACHE II Score	Nonoperative	Postoperative
0–4	4%	1%
5–9	8%	3%
10–14	15%	7%
15–19	25%	12%
20–24	40%	30%
25–29	55%	35%
30–34	73%	73%
>34	85%	88%

**Table 4:** Model performance (AUC).

Risk Score Calculators	Model Performance (AUC)
RCRI Index (Revised Cardiac Risk Index)	0.70–0.80
AUB-HAS2 (The American University of Beirut)	0.70–0.75
APACHE II (Acute Physiology and Chronic Health Evaluation II)	0.75–0.85
POSSUM (Physiological and Operative Severity Score for the enumeration of Mortality and Morbidity)	0.70–0.80

**Table 5:** Bleeding risk (adapted according to the ESC 2022 guide).

Minor Bleeding Risk	Low Bleeding Risk	High Bleeding Risk
Cataract or glaucoma procedure Dental procedures: extractions (1–3 teeth), periodontal surgery, implant positioning, endodontic procedures, subgingival scaling/cleaning Endoscopy without biopsy or resection Superficial surgery	Abdominal surgery: cholecystectomy, hernia repair, colon resection Breast surgery Complex dental procedures (multiple tooth extractions) Endoscopy with simple biopsy Gastroscopy or colonoscopy with simple biopsy Large-bore needles procedures Non-cataract ophthalmic surgery Small orthopedic surgery (foot, hand arthroscopy)	Abdominal surgery with liver biopsy, extracorporeal shockwave lithotripsy Extensive cancer surgery Neuraxial anesthesia Neurosurgery Major orthopedic surgery Procedures with vascular organ biopsy (kidney or prostate) Reconstructive plastic surgery Specific interventions (colon polypectomy, lumbar puncture) Thoracic surgery, lung resection surgery Urological surgery Vascular surgery

**Table 6:**
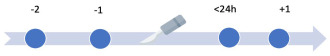
Perioperative management of direct oral anticoagulants-ESC.

	NCS Minor Risk	NCS Low Risk	NCS High Risk
Dabigatran, Apixaban	May skip the evening dose −1 day and restart 6h after surgery	Skip DOAC −1 day and restart in the evening	Skip DOAC −2 days and restart 48–72 h after surgery *
Rivaroxaban, Edoxaban	Normal	Skip DOAC −1 day and restart in the evening	Skip DOAC −2 days and restart 48–72 h after surgery *
Dabigatran EGFR < 50 mL/min	Normal	Stop > 48 h	Stop > 96 h
Apixaban, Edoxaban, Rivaroxaban, EGFR < 50 mL/min	Normal	Stop > 24 h	Stop > 48 h

* Consider prophylactic dose of heparin perioperatively; NCS = non cardiac surgery; DOAC: direct oral anticoagulants, EGFR = estimated glomerular filtration rate.

**Table 7:**
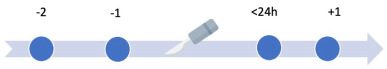
Perioperative management of direct oral anticoagulants-AHA/ACC.

	NCS Minor Risk	NCS Low Risk	NCS High Risk
Apixaban, Edoxaban, Rivaroxaban	Normal	Skip DOAC −1 day and restart +1 day	Skip DOAC −2 day and restart +3 day
Dabigatran	Normal	Skip DOAC −1 day and restart +1 day	Skip DOAC −2 day and restart +3 day
Apixaban, Edoxaban, Rivaroxaban, EGFR < 50 mL/min	Normal	Skip DOAC −2 day and restart +1 day	Skip DOAC −3 day and restart +3 day
Dabigatran EGFR < 50 mL/min	Normal	Skip DOAC −2 day and restart +1 day	Skip DOAC −4 day and restart +3 day

NCS = non cardiac surgery; DOAC: direct oral anticoagulants, EGFR = estimated glomerular filtration rate.

**Table 8:**
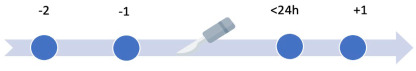
Perioperative management of direct oral anticoagulants-CCS.

	NCS Minor Risk	NCS Low Risk	NCS High Risk
Apixaban, Edoxaban, Rivaroxaban, Dabigatran	Normal	Skip DOAC −1 day and restart 3–4 h post-operative	Skip DOAC −2 day and restart +2 day
Dabigatran EGFR < 50 mL/min	Normal	Skip DOAC −2 day and restart 3–4 h post-operative	Skip DOAC −4 day and restart +2 day

NCS = non cardiac surgery; DOAC: direct oral anticoagulants, EGFR = estimated glomerular filtration rate.

**Table 9:** Main recommendations/Gaps in evidence.

Main Recommendations	Gaps in Evidence
An adequate pre-operative risk assessment may reduce the risk of complications; Clinical examination is at least as important as paraclinical investigation; Selection of type and time of the intervention is essential; TTE is recommended before NCS on patients with sign or symptoms of CVD and elevated NT-pro BNP/BNP Apirin should be stopped 7 days before the procedure on patient with high bleeding risk; Patients on DAPT can stop P2Y12 after 6 months for elective PCI and at 12 months for acute coronary syndrome; Surgeries with minimal bleeding risk should be performed without interrupting oral anticoagulation therapy; Bridging with heparin is necessary only on patients with high bleeding risk and mechanical prosthetic heart; Routine initiation of beta-blockers peri-operatively is not recommended.	A more precise instrument for calculating perioperative risk; Studies to better evaluate gender inequalities in risk assessment; Strategies to reduce the incidence of peri-operative atrial fibrillation Studies for indication of anticoagulation of peri-operative atrial fibrillation; Insufficient studies for antihypertensive medication, comparisons between drugs, and long-term safety; Evaluation of patients with severe pulmonary hypertension who undergo NCS.
